# Satisfactory Functional Outcome and Significant Correlation with the Length of Haglund's Deformity after Endoscopic Calcaneoplasty: A Minimum 4-Year Follow-Up Study

**DOI:** 10.1155/2022/7889684

**Published:** 2022-04-13

**Authors:** Hossam Fathi Mahmoud, Walid Feisal, Fahmy Samir Fahmy

**Affiliations:** Department of Orthopedic Surgery, Faculty of Medicine, Zagazig University, Zagazig, Egypt

## Abstract

**Background:**

Haglund's syndrome is a posterosuperior calcaneal prominence with posterior heel pain causing functional disability to the patient. Operative treatment is indicated after failure of conservative measures and includes resection of the bony hump along with retrocalcaneal bursectomy. This study aimed to evaluate the functional outcome and degree of patient satisfaction after endoscopic resection of Haglund's deformity with assessment of correlation with the length of bony exostosis.

**Methods:**

Seventeen patients (21 feet) with a mean age of 44.7 ± 5.1 years were included in this study. Six females and 11 males underwent endoscopic calcaneoplasty. Clinical outcome evaluation included the assessment of the visual analog scale (VAS) and the American Orthopedic Foot and Ankle Society score (AOFAS). The preoperative and postoperative lengths of bony exostosis were measured radiologically. The paired *t* test and the Wilcoxon signed-rank test compared the preoperative and final postoperative means. *P* < 0.05 was considered statistically significant.

**Results:**

The mean follow-up period was 56.4 ± 5.1 months. Statistically significant improvements in the preoperative mean of AOFAS (from 55.7 ± 9.3 to 94.3 ± 7.1) and VAS (from 8.1 ± 1.4 to 0.7 ± 1.04) at the final follow-up were noted (*P* < 0.0001). There was a statistically significant correlation between clinical scores (AOFAS and VAS) and the final postoperative length of the bone above parallel pitch lines (PPLs). The patients were satisfied and returned to their previous activities without reporting major morbidities except one patient who had mild pain with exertion.

**Conclusion:**

Endoscopic calcaneoplasty is a safe, less invasive surgical procedure for the management of Haglund's syndrome after failure of conservative treatment. It provided a satisfactory clinical result without serious complications at a minimum 4-year follow-up.

## 1. Introduction

Patrick Haglund, a surgeon, first described Haglund's syndrome as a prominence of the posterosuperior calcaneal tuberosity resulting in disabling posterior heel pain. Para tendinitis, insertional Achilles tendinosis, retrocalcaneal bursitis, and bony exostosis with persistent swelling adjacent to the Achilles tendon insertion are the main pathological findings [1–3].

The incidence is more common in females in their late 30s with bilateral affection [4]. The primary cause is idiopathic; nevertheless, certain contributing factors influence the condition including varus heel, hindfoot equinus, tight or poorly fitted shoes, and runners due to repeated overuse [5].

The chief complaints are posterior heel pain, swelling, and limping. The main clinical findings are tender posterior bony hump with exacerbation of pain during active and passive ankle dorsiflexion. Radiological diagnosis is made on lateral x-ray view to detect the prominence of posterosuperior calcaneal tuberosity. Magnetic resonance imaging (MRI) is helpful to identify bursal thickening, Achilles tendinosis, and signal changes in the bony insertion [6, 7].

Conservative management is the mainstay of treatment, which includes activity level modification, heel elevation using cushion in shoes, nonsteroidal anti-inflammatory drugs (NSAIDs), local platelet-rich plasma injection, and ultrasound device. Extracorporeal shock wave therapy reduces pain and achieves 80% of patient satisfaction [8]. Conservative management is successful in 85%–95% of patients [9].

Operative treatment is indicated after the failure of conservative treatment for at least 6 months. The goal of surgery was to remove the posterosuperior bony exostosis and debridement of the inflamed bursa. Open surgical resection is widely used among orthopedic surgeons; however, it had some complications, such as wound dehiscence, Achilles tendon avulsion, ugly scar, persistent heel pain, medial calcaneal sensory nerve lesion, sural nerve injury, painful neuroma, and prolonged immobilization and recovery [10–12].

The endoscopic procedure is superior to open surgery because of the lower postoperative complications, early mobilization, faster rehabilitation, and rapid return to previous activities. The endoscopic technique provides excellent medial and lateral side visualization of the retrocalcaneal space especially at the Achilles tendon bony insertion [13].

This study aimed to evaluate the functional outcome and patient satisfaction after endoscopic calcaneoplasty with a minimum 4-year follow-up. Moreover, we assessed the correlation of the clinical scores with the length of bony exostosis. We hypothesized that significant improvements in the postoperative clinical scores with low complications were observed.

## 2. Patients and Methods

Our ethical institutional review board approved this study (ZU-IRB # 988/22-1-2016), and all patients waived informed consent for participation. Patients who suffered from chronic posterior heel pain due to Haglund's deformity underwent endoscopic calcaneoplasty between January 2016 and January 2022 at Zagazig University Hospitals. The patients had conservative treatment in the form of rest, physical therapy, NSAIDs, shock wave therapy, and local platelet-rich plasma injection for at least 6 months preoperatively. The criterion for inclusion was chronic heel pain (>6 months) refractory to conservative treatment. Patients with previous ankle or calcaneal fractures, painful insertional calcification, seronegative arthritis, ankle deformities, Achilles tendon degeneration >50% of thickness, and skeletally immature ankles were excluded from the study.  Table 1 presents the demographic data of the patients.

**Figure 1:**
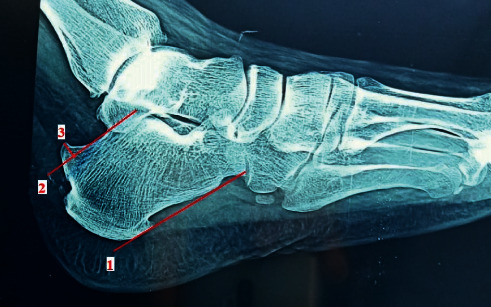
Length of bony exostosis is measured on lateral X-ray view of the calcaneus. Line 1 starts from the most inferior point of the calcaneocuboid along the plantar aspect of the calcaneus. Line 2 extends from the highest point of the subtalar and is parallel to line 1. Line 3 indicates the length of the bony hump above line 2.

**Figure 2:**
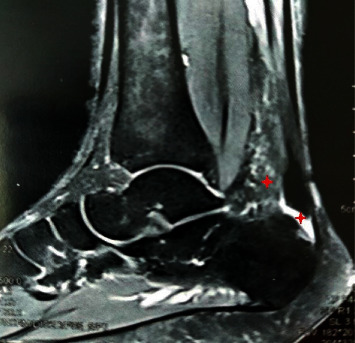
Magnetic resonance imaging T_2_ sequence, sagittal view showing an increased signal intensity of the retrocalcaneal bursa with marked fibrosis anterior to the Achilles tendon (red asterisks).

All patients underwent clinical assessment through history taking, including the activity level, physical examination, and provocative tests preoperatively. The patients had swelling and tenderness at the sides of the Achilles tendon insertion. Pain was provoked by active and passive ankle extension. To assess the Achilles tendon integrity, we used the Thompson calf squeeze test. The length of bony exostosis above the parallel pitch lines (PPLs) was measured on lateral x-ray view of the calcaneus (Figure 1). MRI assessed the degree of tendon degeneration and presence of inflamed retrocalcaneal bursa (Figure 2). The visual analog scale (VAS) [14] and American Orthopedic Foot and Ankle Society (AOFAS) scores [15] were reported for the functional assessment.

### 2.1. Surgery

**Figure 3:**
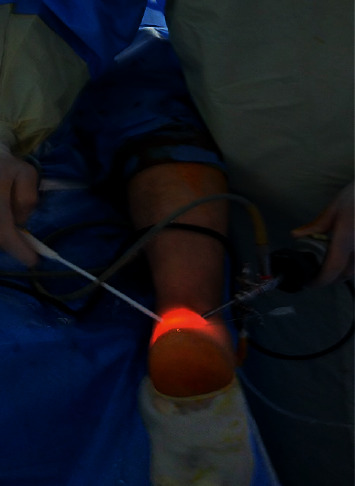
Patient is lying prone intraoperatively. The procedure was performed through medial and lateral portals.

**Figure 4:**
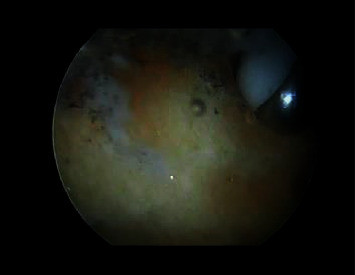
Clearance of the soft tissue to facilitate the resection of the bony prominence using a radiofrequency ablation device.

**Figure 5:**
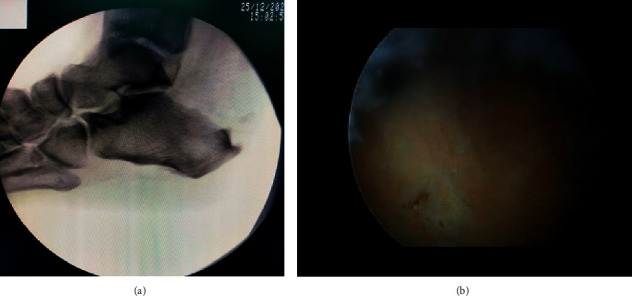
Intraoperative fluoroscopic (a) and arthroscopic (b) images showing complete resection of the excess bone.

An intravenous antibiotic (cefotaxime 1 gm) was administered 30 minutes preoperatively. The patient was positioned prone with the foot hanging over the edge of the operating table (Figure 3). Surgeries were performed with a well-padded tourniquet tied over the thigh. The procedure was performed through two portals placed just medial and lateral to the Achilles tendon and above the superior part of the calcaneus. The portals were interchanged for viewing and working. A 4-mm motorized shaver was used to remove the retrocalcaneal bursa and degenerated parts of the tendon. The bony exostosis was cleared from soft tissue with the aid of a radiofrequency ablation device (Figure 4). Subsequently, the surgeon removed the excess bone with a 3.5-mm motorized burr until the tendon did not impinge with the posterosuperior part of the calcaneal tuberosity during ankle dorsiflexion. The medial and lateral edges of the bone were carefully inspected to avoid leaving any bony prominence. The amount of bone resection was judged intraoperatively using a C-arm image intensifier (Figure 5). Finally, the portals were stitched.

**Figure 6:**
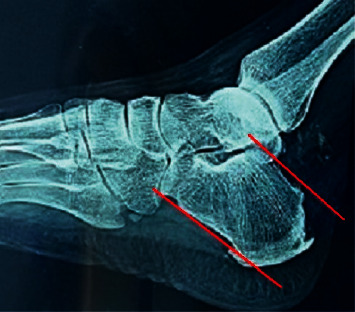
Adequate endoscopic resection of the bone with no prominence above the parallel pitch line (red line) at the 4-year postoperative follow-up.

The antibiotic was continued for 48 hours, and the stitches were removed after 14 days postoperatively. The patients were instructed to elevate the limb during the first postoperative week. Active and passive motions were started immediately postoperative. Partial weight bearing was allowed in the first 2 weeks and full weight bearing after the third week. The patients returned to daily activities 6–8 weeks after the surgery. The clinical scores (AOFAS and VAS) and mean radiological length of the bony hump were recorded at the end of follow-up (Figure 6).

### 2.2. Statistical Analysis

The distribution of data was tested using the Kolmogorov–Smirnov test. Numerical data were presented as percentages and means with standard deviation. Normally distributed preoperative and final postoperative means were compared using the paired *t* test, and nonparametric means were compared using the Wilcoxon signed-rank test. The postoperative length of the bony hump and final clinical scores were correlated using Pearson′s coefficient. A *P* value of < 0.05 was considered statistically significant. A priori sample size for the two-sided matched paired *t* test that provided 85% statistical power and large size difference at 0.05 *α* error was estimated using G-Power software calculator version 3.1. The Statistical Package for Social Sciences (SPSS) data editor version 16 (SPSS for Windows, version 16.0.; SPSS Inc., Chicago, IL, USA) was used for statistical analysis.

## 3. Results

Seventeen patients (21 heels) with a mean age of 44.7 ± 5.1 (36–52) years underwent endoscopic calcaneoplasty for Haglund's deformity by the same surgeon. The mean duration of symptoms was 10.7 ± 2.6 (7–16) months. The mean time of operation was 52.3 ± 12.3 (35–75) minutes. The mean follow-up time was 56.4 ± 5.1 (48–64) months. No patients were missed during the follow-up.

The AOFAS ankle-hindfoot score increased from 55.7 ± 9.3 (37–68) preoperatively to 94.3 ± 7.1 (72–100) postoperatively with statistically significant improvement (*P* < 0.00001) at the final follow-up. According to AOFAS, 12 patients had excellent (90–100 points), 4 good (80–89 points), and 1 fair (70–79 points) clinical results, respectively.

The VAS for pain improved from 8.1 ± 1.4 (6–10) preoperatively to 0.7 ± 1.04 (0–3) postoperatively. The means were compared using the Wilcoxon signed-rank test (*P* < 0.00001) (Table 2).

The preoperative and postoperative mean length of bony prominence above the PPL on lateral radiographs were recorded and compared. The preoperative mean improved from 4.7 ± 1.4 (3–7 mm) to −0.2 ± 1.1 (−2 to 2 mm) at the final follow-up with a significant statistical difference (*P* < 0.00001) (Table 2).

We found a significant negative correlation between the final mean length of the bony hump and the final AOFAS score (*R* = −0.7; *P*=0.0004) and a positive correlation with the final VAS for pain (*R* = 0.63; *P*=0.002) using Pearson's coefficient.

The patients returned to work and regained their previous activity level with a mean time of 5.9 ± 1.6 weeks. They were satisfied with the results except for one patient who developed mild postoperative pain with strenuous activities.

No major complications were reported; however, one patient developed transient postoperative sural nerve neuropraxia that was resolved within 3 months.

## 4. Discussion

The main finding of the study was statistically significant improvements in the functional scores (AOFAS ankle-hindfoot and VAS for pain) (*P* < 0.00001). Ninety-four percent of patients were satisfied and resumed their preoperative activities with a mean follow-up of 56.4 ± 5.1 months.

Haglund's syndrome is a common cause of posterior heel pain. The enlarged posterosuperior calcaneal tuberosity impinges on the retrocalcaneal bursa with attrition of the ventral surface of the Achilles tendon during repeated foot extension, which results in retrocalcaneal bursitis, Achilles tendon degeneration, and concomitant pathological tendon rupture. The cornerstone of surgical management is adequate exostosis resection and retrocalcaneal bursa debridement without violation of the Achilles tendon insertion site. The rationale of surgical treatment can be achieved by open or arthroscopic techniques [16].

Leitze et al. [17], Cusumano [11], and Chimenti [18] advocated the endoscopic technique over open surgery, owing to minimal incision, higher degree of patient satisfaction, and lower morbidities. Patients who underwent the endoscopic technique can expect less pain postoperatively, thereby permitting an earlier return to work, and sport activities with a lower risk of complications.

Several literatures reported that failure of conservative treatment with a prolonged symptom duration of >6 months was the main indication of surgical intervention [8, 9]. We reported a mean duration of 10.7 ± 2.6 months before surgical management. Moreover, Wu et al. [19] estimated a mean symptom duration of 14.9 months preoperatively.

A study by Jerosch et al. [4] included 81 patients who suffered from Haglund's syndrome; they found that the incidence was more in female patients (41 females and 40 males). This was consistent with the previously published literature that reported a greater incidence among females between 20 and 30 years old [20]. In our study, we found that young males were more affected than females because of engagement in heavy works and strenuous activities. They were also seeking for early return to work through surgical management. Our findings were consistent with the results of Pi et al. [21] (11 females vs. 36 males).

The mean operative time in our study was 52 ± 12.3 minutes, and it decreased from 75 minutes preoperatively to 35 minutes postoperatively. Our mean operative time was shorter than that recorded by Pi et al. [21] (65.4 ± 11.1 minutes). This may be attributed to the small number of patients enrolled in our study (17 vs. 27 patients). Our findings matched with the reported operative time by Kaynak et al. [22] (decreased from 90 to 30 minutes at the last surgery). Additionally, Leitze et al. [17], Wu et al. [19], and Jerosch [4] stated that there was a steep decrease in operative time because of surgical skill improvement (from 120 to 30 minutes, 45 to 20 minutes, and 46 to 35 minutes, respectively).

The final AOFAS score in this study was close to the results of Pi et al. [21] who reported a postoperative mean AOFAS score of 92.1 ± 8 and were consistent with the results of Cusumano et al. [11] (improvement in AOFAS mean score from 66.19 ± 7.19 preoperatively to 93.69 ± 9.67 postoperatively) (*P* < 0.01). Furthermore, our results were comparable with those of Kaynak et al. [22] and Ortmann [23] who recorded a significant difference between the preoperative and postoperative mean AFOAS scores (from 52.6 preoperatively to 98.6 postoperatively and from 62 preoperatively to 97 postoperatively, respectively) (*P* < 0.01).

The clinical results were graded as excellent (90–100 points), good (80–89 points), fair (70–79 points), and poor (<70 points) according to the AOFAS of the ankle-hindfoot score [15]. We reported final clinical results of 12 excellent, 4 good, and 1 fair; no poor results were found at the end of follow-up. Our results were better than those recorded by Wu et al. [19] (14 excellent, 7 good, 2 fair, and 2 poor clinical results, with a final postoperative mean of 86.8 ± 10.1 points) and Leitze et al. [17] (19 excellent, 5 good, 3 fair, and 3 poor clinical results, with an average of 87.5 points at the end of follow-up).

Regarding the VAS for pain, our results were comparable with those of Pi et al. [21] who reported a final postoperative mean VAS of 0.9 ± 1.2, and it was better than that recorded by Cusumano et al. [11] who showed improvement in the VAS from 7.57 ± 1.27 preoperatively to 1.30 ± 1.93 postoperatively.

We did not find a significant difference between our results and the final postoperative means of AOFAS and VAS scores that were reported in 20 patients who underwent open surgery by Pi et al. [21]. Their final means were 96.1 ± 5.1 and 0.9 ± 1.2 for AOFAS and VAS scores, respectively. Leitze et al. [17] did not report a statistically significant difference in the final postoperative mean of the AOFAS score between open (79.3 points) and endoscopic surgery (87.5 points) (*P*=0.115).

An adequate resection of the bony hump intraoperatively is essential to avoid postoperative recurrence of symptoms with no consensus about the ideal radiographic measurement to judge the amount of resection [19,24]. We used Pavlov's PPL as a radiological reference to estimate the amount of bony resection. We had four positive heels with a range of 1–2 mm above Pavlov's PPL, and 17 heels were negative (−2 to 0 mm) with a final postoperative mean of −0.2 ± 1.1 mm. Wu et al. [19] recorded a negative bone length above PPL in 19 patients, and only three heels were positive. Our results were better than those of Pi et al. [21] who reported a postoperative mean length of Haglund's deformity of 5.0 ± 1.6 mm. Opdam et al. [3] recorded a median 1 mm bone length (0–2 mm) at the end of follow-up.

We found a statistically significant correlation between the functional scores (AOFAS and VAS) and the postoperative length of the bony hump. We recorded a negative correlation with AOFAS (*R* = −0.7) (*P*=0.0004) and a positive correlation with VAS (*R* = 0.63) (*P*=0.002), which were contrary to the previously reported results of Opdam et al. [3] and Wu et al. [19] who estimated good clinical results of AOFAS in three heels, which were positive regarding the bone length above PPL.

The mean time of return to work was 5.9 ± 1.6 weeks, which was comparable with the result of Atanu et al. [1] who stated that the average time of regaining the previous activity level was 6 (4–8) weeks. Kaynak et al. [22] allowed sports training for their patients 6 weeks postoperatively.

We found two minor complications (11.8%); one patient developed transient sensory sural nerve neuropraxia and the other, a female housewife, who developed mild pain with heavy exertion without limitations of daily activities. All patients regained their previous activity level, and 94.1% of patients were satisfied with the results. Similar results were recorded by Leitze et al. [17] who had one patient with mild sural neuropathy and two patients with residual tenderness. Scholten and Van Dijk [25] recorded one patient with mild sensory hypoesthesia of the heel and two with persistent discomfort with an 8.3% complication rate. Angermann [26] and Huber and Waldis [27] reported major skin and wound healing complications in their patients who underwent open calcaneoplasty (10% and 43.7%, respectively).

Our study had some limitations. First, the number of included patients was small; however, it was enough based on the estimated power analysis, although a larger sample size is recommended for the generalizability of the results and conclusions. Second, there was no control group for comparison with other treatment options. Additionally, the endoscopic procedure needed a well-trained surgeon with a stepwise improvement of the learning curve. Finally, the healing process was not investigated.

## 5. Conclusion

Endoscopic calcaneal resection of Haglund's deformity provided improvement in the clinical scores (AOFAS and VAS) with minor complications at a minimum 4-year follow-up. Approximately 94% of patients were satisfied and regained their previous activity level. Small wound, early recovery, direct visualization, and controlled bone resection are the main advantages of the endoscopic technique. We found a statistically significant correlation between the functional scores and the radiological length of bony exostosis. Further studies with a larger number of patients will be needed in the future to prove this correlation and investigate the nature of healing.

## Figures and Tables

**Table 1:** Demographic characteristics of the patients.

Characteristic	Mean ± SD (range) or *n* (%)
Age (years)	44.7 ± 5.1 (36–52 years)
Sex	
Male	11 (64.7%)
Female	6 (35.3%)
Affected side	
Right	8 (47.1%)
Left	5 (29.4%)
Bilateral	4 (23.5%)
Symptom duration	10.7 ± 2.6 (7–16 months).
Occupation	
Farmer	3 (17.6%)
Basketball trainer	1 (5.9%)
Football player	1 (5.9%)
Employee	4 (23.5%)
Factory worker	4(23.5%)
Housewives	4 (23.5%)

*N*: number, SD: standard deviation, and %: percent. Numerical data are presented as mean ± SD (range) and percentage.

**Table 2:** Final results of functional scores and radiological measurement.

	Preoperative mean	Final postoperative mean	*P* Value
AOFAS ankle-hindfoot score	55.7 ± 9.3 (37–68)	94.3 ± 7.1 (72–100)	*P* < 0.00001
VAS for pain	8.1 ± 1.4 (6–10)	0.7 ± 1.04 (0–3)	*P* < 0.00001
Radiological length of Haglundʼs deformity	4.7 ± 1.4 (3–7 mm)	−0.2 ± 1.1 (−2 to 2 mm)	*P* < 0.00001

AOFAS: American Orthopedic Foot and Ankle Society, VAS: visual analog scale, and mm: millimeter. *P* < 0.01 was considered highly significant.

## Data Availability

Data used to support the findings of this study are available from the corresponding author upon request.
